# The Immunomodulatory Effects of a 6-Month Extra Virgin Olive Oil Intervention on Monocyte Cytokine Secretion and Plasma Cytokine Levels in Dyslipidemic and Post-Infarct Patients: A Clinical Pilot Study

**DOI:** 10.3390/nu15173819

**Published:** 2023-08-31

**Authors:** Adrien Zimmer, Alyann Otrante, Nada Zoubdane, Michel Nguyen, Tamàs Fülöp, Abdelouahed Khalil

**Affiliations:** 1 Geriatrics Unit, Department of Medicine, Faculty of Medicine and Biological Sciences, University of Sherbrooke, Sherbrooke, QC J1H 4N4, Canada; adrien.zimmer@usherbrooke.ca (A.Z.); alyann.otrante@usherbrooke.ca (A.O.); zoubdane.nada@usherbrooke.ca (N.Z.); tamas.fulop@usherbrooke.ca (T.F.); 2 Cardiology Unit, Department of Medicine, Faculty of Medicine and Biological Sciences, University of Sherbrooke, Sherbrooke, QC J1H 4N4, Canada; michel.nguyen@usherbrooke.ca; 3 Research Center on Aging, Integrated University Center for Health and Social Services of Estrie—University Hospital Center of Sherbrooke, Sherbrooke, QC J1H 4N4, Canada; 4 Research Center of the Centre Hospitalier Universitaire de Sherbrooke, Sherbrooke, QC J1H 4N4, Canada

**Keywords:** atherosclerosis, hypercholesterolemia, infarcts, EVOO, monocyte, inflammation

## Abstract

Atherosclerosis is an immuno-inflammatory process underlying cardiovascular diseases. One of the main actors of this inflammation is monocytes, with the switch in their phenotypes and irregularities in their cytokine production. Objective: This study was aimed to investigate the nutraceutical potential of extra virgin olive oil (EVOO) on the inflammatory status of monocytes in participants showing different levels of cardiovascular risk. Methods: 43 participants 65–85 years old were recruited including 14 healthy, 12 dyslipidemic patients with hypercholesterolemia recently diagnosed, and 17 post-infarct patients. Participants from all groups were supplemented with EVOO (25 mL/day) for 6 months. IL-1β, IL-6, IL-10, TNF-α cytokine production, and monocyte phenotypes were investigated both at quiescent and at stimulated state by flow cytometry. Results: At the baseline (pre-intervention), dyslipidemic patients, compared to healthy and post-infarct participants, showed monocytes in an inflammatory state characterized by a significantly weaker IL-10 production. Our results do not show a significant modulation of the phenotype or IL-10, IL-6, and TNF-α production following a 6-month EVOO intake whether at quiescence or under stimulation. However, IL-1β is significantly increased by the intervention of EVOO in post-infarct patients. Paradoxically after the 6-month intervention, monocytes from dyslipidemic patients showed a significantly decreased secretion of IL-1β under LPS stimulation despite the increase observed at basal state. Conclusion: Our results show that 6-month EVOO intervention did not induce a monocyte phenotypic change or that this intervention significantly modifies cytokine production.

## 1. Introduction

Atherosclerosis is a chronic inflammatory disease characterized by the accumulation of lipids, inflammatory cells, and extracellular matrix components in the arterial wall. This process can lead to the formation of plaques that can potentially rupture and lead to thrombosis which can hinder or stop blood flow to vital organs and lead to infarcts or stroke. This pathology, with all the treatments available, remains a major cause of morbidity and mortality worldwide. Several risk factors are associated with this disease such as high blood pressure, smoking, and high levels of low-density lipoprotein cholesterol (LDL-C).

Several studies have documented an inverse association between adherence to the Mediterranean diet and risk of coronary heart diseases (CHD) [1,2,3]. Extra virgin olive oil (EVOO) is one of the key components of this diet. It is rich in monounsaturated fatty acids, polyphenols, and other bioactive compounds that have been shown to have antioxidant and anti-inflammatory effects [4]. Interestingly, inflammation, as well as the interaction between cells with the plaque, are the major determinants of plaque progression and instability. Although innate and adaptive immunity actors are all present in advanced plaques, monocytes play a key role in the pathogenesis of atherosclerosis [5]. Monocytes, after being recruited from the bloodstream into the arterial wall, differentiate into macrophages and through their absorption of oxidized lipoproteins become the main contributor to the formation of the lipid/necrotic core. Monocytes and macrophages are also important sources of cytokines that reveal their inflammatory state and influence the fate of the plaques.

Monocytes are a critical component of the cellular innate immune system, and can be subdivided into classical, intermediate, and non-classical subsets based on surface CD14 and CD16 expression. Classical monocytes (high CD14 but no CD16 expression) play the canonical role of phagocytosis, support the inflammatory process, and account for most circulating cells. Intermediate (high CD14 and low CD16 expression) and non-classical cells (relatively lower CD14 expression and high CD16) are known to exhibit varying levels of phagocytosis and cytokine secretion and are differentially expanded in certain pathological states. The intermediate monocytes contribute through antigen presentation to T cell activation and clonal proliferation and produce large amount of ROS while the non-classical monocytes activate CD4+ T cells and patrol the endothelium [6,7].

Some studies revealed that monocytes may be pre-activated in the circulation of CVD-high-risk patients and might be more susceptible to have pro-inflammatory phenotypes predisposing them to become pro-inflammatory macrophages in the plaque, hence promoting its growth and instability [8]. This pre-activated state is called trained immunity, a process demonstrated in vivo notably with oxLDL [9]. In atherosclerosis and dyslipidemia on top of this phenomenon, some studies report that these monocytes could become dysfunctional due to these pathologies and lose their specific pro or anti-inflammatory phenotype and all show pro-inflammatory activity [10].

Several studies have investigated the effect of EVOO on the production of cytokines in monocytes and macrophages. In vitro studies have demonstrated that EVOO and its phenolic compounds can modulate the expression of cytokines such as tumor necrosis factor-alpha (TNF-α), interleukin-1 beta (IL-1β), and interleukin-6 (IL-6) mostly using monocytes—macrophages and THP-1 [11] or J774 models [12].

Animal studies have also suggested that EVOO can reduce the expression of pro-inflammatory cytokines in monocytes and macrophages and attenuate the development of atherosclerosis [13]. EVOO compared to sunflower oil rich-diet group was shown to induce a reduction in the inflammatory cytokine production in C57BL/6 mice [14].

Clinical studies have provided evidence for the anti-inflammatory effects of EVOO in humans. A Mediterranean diet supplemented with EVOO for three months led to a reduction in the expression of adhesion molecules on peripheral blood mononuclear cells as well as a reduction in inflammatory cytokine concentrations in plasma [15,16]. The effect of EVOO combined with a Mediterranean diet compared to low-fat diet showed a beneficial effect on vascular inflammation [17]. However, the effect of EVOO intervention on human monocytes are scarcer although the anti-inflammatory effect of polyphenols in vitro on monocytes/macrophages is clearly established [11,12,18]. This pilot study aimed to investigate the nutraceutical potential of EVOO of 6-month EVOO intervention on monocyte’s inflammatory status depending on the level of cardiovascular risk—dyslipidemic and post-infarct patients compared to healthy control individuals.

## 2. Materials and Methods

### 2.1. Recruitment of Patients

This study was conducted within the framework of the LIPIMAGE cohort, which is an ongoing prospective study using positron emission tomography imaging to investigate the effect of EVOO on atherosclerotic plaque progression and stability in patients at high risk for CVD. The study was conducted according to the guidelines set out in the Declaration of Helsinki. The protocol was approved by the Ethics Committee of the Sherbrooke University Hospital Center (#2019-3145). Written informed consent was obtained from all subjects.

**Figure 1:**
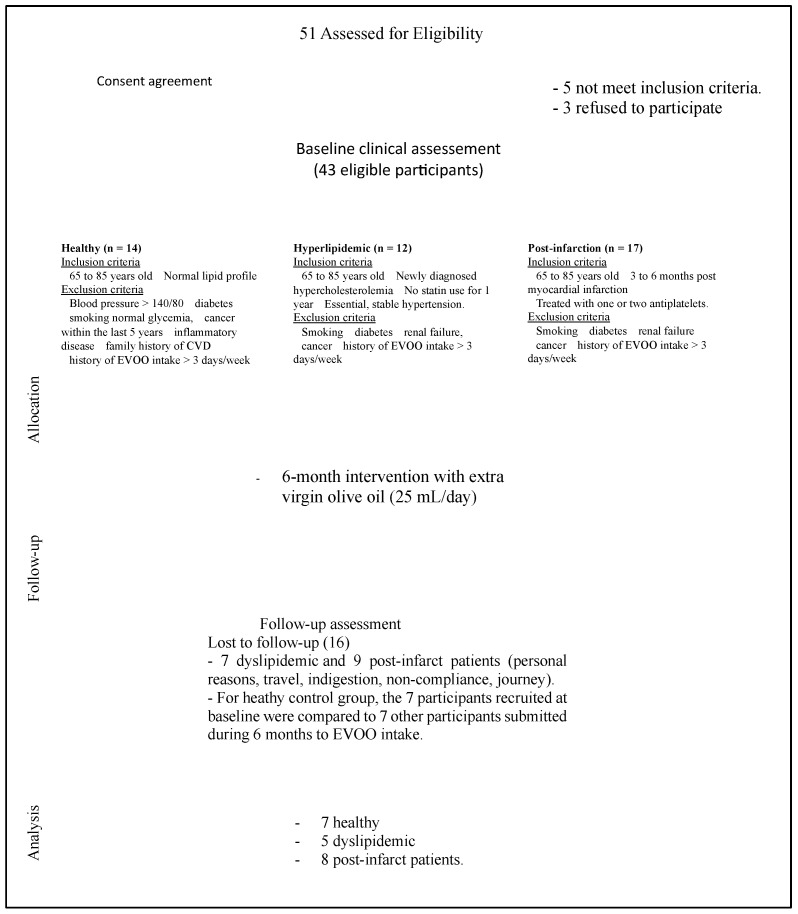
The flowchart of the study.

A total of 43 participants (65–85 years old) were recruited from the LIPIMAGE cohort. Participants were distributed, according to their cardiovascular-risk level, into three groups (Figure 1). Healthy group includes 14 healthy individuals that do not present any recent or familial medical record with normal arterial pressure (PA): 140/85 mmHg and normal lipid profile, a BMI comprised between 23 and 28 kg/m², and exposing a normal ECG. The dyslipidemic group was formed with recently diagnosed dyslipidemic patients (12 patients) who did not receive lipid-lowering therapy throughout their 6-month participation in the study. These patients were selected for having hypercholesterolemia with LDL-C values between 3.5 and 5 mmol/L (excluding patients with familial hypercholesterolemia). The third group encompassed myocardial infarction patients (17 patients) admitted to this study at least 3 months after their infarct event as to stabilize their infarct-associated inflammation.

Exclusion criteria for all participants were tobacco use, diabetes (HbA1c > 6%), chronic inflammatory diseases, kidney failure, collagenases, or cancer carriers, taking anti-inflammatories, omega-3 fatty acids, or replacement hormones for women. Participants also taking EVOO on a usual basis (>3 times a week and in raw form) were also excluded.

Participants were asked to consume 25 mL/day of EVOO and compliance to the intervention was checked with monthly appointments where a questionnaire was issued and total EVOO consumed could be assessed as they gave back previous EVOO containers. No specific recommendations regarding diet or physical activity before the study were given to the participants. All subjects normally participated in all their daily activities without modifications throughout the study duration. Blood collection for experiments and blood tests were performed at recruitment and after 6 months of EVOO consumption. During the 6 months of intervention, some participants dropped out of the study (7 dyslipidemic and 9 post-infarction patients) for personal reasons, change of medication, problem of compliance, or long journey. For healthy participants, we compared data obtained from participants at the baseline to those obtained from the same number of other healthy participants who were selected according to the same inclusion and exclusion criteria and who were subjected to an EVOO-rich diet (25 mL/day for 6 months).

### 2.2. Extraction of Plasma and Purification of Monocytes

Patients’ blood was collected in heparin tubes, after which quick centrifugation (400× *g* for 15 min) allowed to extract plasma. The PBMC (Peripheral Blood Mononuclear Cells) were then separated from the red blood cells using the Ficoll-Hypaque density gradient centrifugation, followed by the separation of monocytes from lymphocytes on a high-density hyper-osmotic Percoll density gradient according to Menck et al. method [19]. The isolated monocytes were then incubated at 37 °C in a humid environment with 5% CO_2_ in 12-well plates with RPMI 1640 supplemented with 10% FBS and 1% antibiotic-antimitotic. The monocytes were stimulated for 4 h with 100 ng/mL LPS (lipopolysaccharide).

### 2.3. FACScan Analysis

The FACS staining was performed according to the manufacturer’s instructions (BD bioscience). The FACScan machine used was a Cytoflex 30 (laser UV) B5-R3-V3-NUV2. Cells were treated with 1 mL Brefeldin A (ab51-2301KZ) simultaneously with LPS stimulation. After 4 h, extracellular staining was achieved using CD14 (ab557742) and CD16 (ab556618), whereas intracellular cytokine [IL-1β (ab340516), IL-6 (ab563279), TNF-α (ab566957), IL-10 (ab554707)] staining was performed after cells fixation and permeabilization (ab555028). The gating strategy used for identifying and separating monocyte subpopulation is presented in Figure S1 (Supplementary Data).

### 2.4. Plasma Analysis

Cytokine levels in the plasma was assessed using a Luminex with a Human High Sensitivity Kit #HSTCMAG-28SK-12 from Millipore Sigma, Burlington, MA, USA.

### 2.5. Statistical Analysis

Per-protocol analysis was used. After assessing normality using the Shapiro–Wilk test, the results were consequently analyzed using paired *t*-test, Wilcoxon test, or impaired *t*-test (for healthy groups) between pre- and post-intervention. ANOVA, unpaired *t*-test, and Mann–Whitney test were performed to compare the different health status between groups before and after 6 months of EVOO intervention. A *p*-value less than 0.05 (typically ≤ 0.05) was considered significant. Statistical analyses were conducted on GraphPad Prism version 9.5.1.

## 3. Results

### 3.1. Study Population

Table 1 present the demographic and clinical parameters of participants from the three groups. The selected groups were comparable in their demographic and clinical parameters and the only exception was the lipid parameters (total cholesterol, HDL-C, LDL-C, non-HDL-C). Dyslipidemic patients present, at baseline, significantly higher total cholesterol level (6.01 +/− 0.42, *p* < 0,05), higher LDL-C (3.56 +/− 0.38, *p* < 0,05), and higher non-HDL cholesterol (4.27 +/− 0.48, *p* < 0,05) when compared to both healthy and post-infarct patients (Table 1). These values underline the clinical differences between the heathy and dyslipidemia subjects (Table 1). It is of note that the creatinine levels were also significantly different but remained in the normal value range. Table 1 also presents the demographic and clinical parameters of the three studied groups after the 6-month EVOO intervention. The intervention did not significantly affect the lipid profile of these three groups to the exception of ALT which remained in normal ranges.

### 3.2. Monocyte Subpopulation Distribution and Polarization by LPS and EVOO Effect

**Figure 2:**
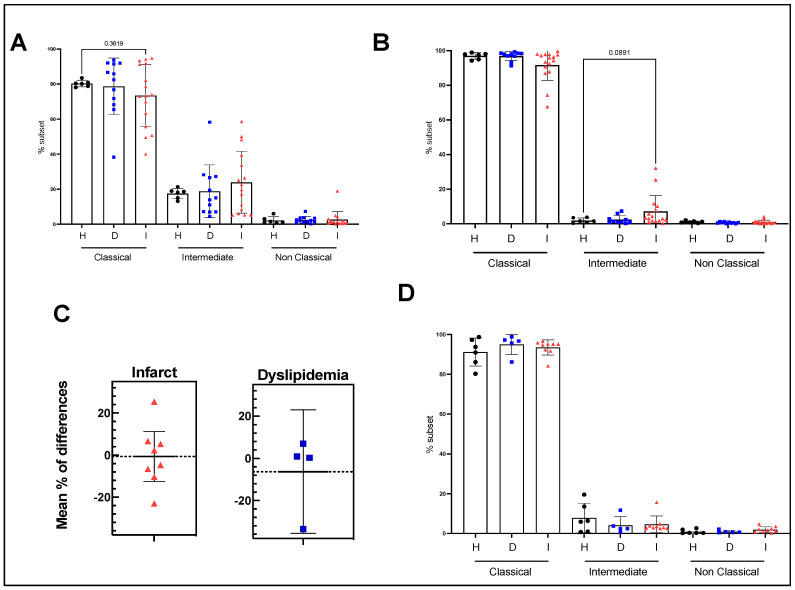
Monocyte sub-population distribution (%) across health status at quiescent state and polarization of these subsets with LPS and effect of EVOO on these parameters. (**A**) Distribution of monocyte subpopulation at quiescent state by health status. (**B**) Distribution of monocyte subpopulation after 4 h of LPS stimulation for each condition before intervention. (**C**) Difference in classical monocyte (%) between pre and post EVOO intervention for dyslipidemia and post-infarct patients. (**D**) Distribution of monocyte subpopulation after 4 h of LPS stimulation for each condition after EVOO intervention. H: healthy participants (*n* = 7 before and 7 after). D: dyslipidemic patients (*n* = 12 before and 5 after) and I: post-infarct patients (*n* = 17 before and 8 after).

First, we determined the monocyte subpopulations (classical, intermediate, and non-classical) in the three groups of subjects. Our data do not indicate any significant difference in the monocyte subpopulation distribution between the three groups (Figure 2A). When the polarization in classical monocyte was induced by LPS, we found that all three groups of patients were able to similarly shift their monocyte subsets into the classical phenotype without any significant discrepancy among the groups (Figure 2B). Interestingly, 6-month EVOO intervention did not induce any significant changes in monocyte distribution whether it be at quiescence and under LPS stimulation (Figure 2C,D)**.**

### 3.3. Production of Cytokines by Monocyte Sub-Populations with and without Stimulation and Effect of EVOO Intervention

**Figure 3:**
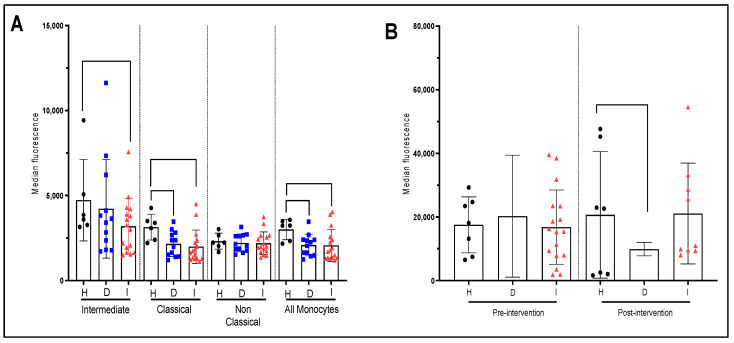
Production of IL-1β by monocyte sub-populations with and without LPS stimulation and the effect of EVOO intervention. (**A**) Pre-intervention cytokine production by monocyte sub-population at quiescent state for each patient groups. (**B**) Pre-and post-intervention cytokine production for the whole monocyte population after 4 h of LPS stimulation for each patient’s group. The values in the figure represent the measurement of the median fluorescence for IL-1β. H: healthy participants (*n* = 7 before and 7 after). D: dyslipidemic patients (*n* = 12 before and 5 after) and I: post-infarct patients (*n* = 17 before and 8 after).

We subsequently determined the production of IL-1β in various monocyte subpopulations in the three subject’s groups. Our results showed significant differences in the production of IL-1β between the three groups. Monocytes from dyslipidemic patients produce significantly less IL1-β compared to healthy subjects (*p* < 0.05) (Figure 3A). Furthermore, the post-infarct patients also produce less IL1-β than healthy subjects (*p* < 0.05). We could not find significant differences between dyslipidemic and post-infarct patients with respect to IL-1β production. When we consider the production of IL1β by subpopulations, we found that only post-infarct patients showed a significant reduction in IL1-β secretion in intermediate and almost significant in classical monocytes (Figure 3A).

All monocytes, regardless of the donor’s health status (healthy, dyslipidemic, or post-infarct) increased the production of inflammatory cytokines (IL-1β) when stimulated with LPS with variability in the scale of the increase. When we consider the IL-1β production after LPS stimulation in the three groups of participants, our data do not show significant differences between groups at baseline (Figure 3B). However, after 6-month intervention with EVOO, dyslipidemic patients presented a significant decrease in IL-1β production compared to healthy subjects (Figure 3B). Our results also showed that only post infarct patients significantly increased IL-1β production in the intermediate subset (+68% average increase in median fluorescence, *p* < 0.05) and in the whole monocyte population (+47% in average increase in median fluorescence, *p* < 0.05) following 6 months with EVOO intervention (Figure S2). No significant changes were observed in other subsets suggesting that the significant increase in IL-1β in whole monocyte population is due solely to intermediate monocyte activity.

**Figure 4:**
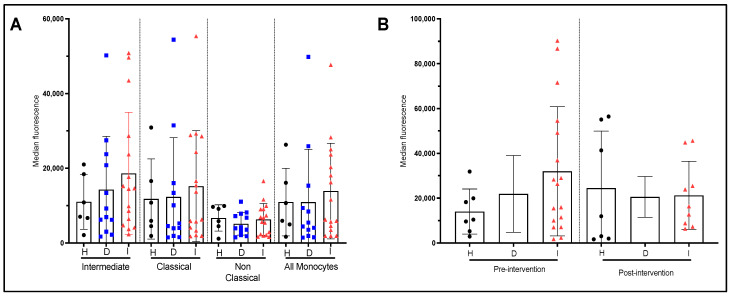
Production of IL-6 by monocyte sub-populations with and without LPS stimulation and effect of EVOO intervention. (**A**) Pre-intervention cytokine production by monocyte sub-populations at quiescent state for each patient group. (**B**) Pre-and post-intervention cytokine production for the whole monocyte population after 4 h of LPS stimulation for each patient/group. The presented values correspond to the measurement of the median fluorescence for IL-6. H: (*n* = 7 before and 7 after). D: dyslipidemic patients (*n* = 12 before and 5 after) and I: post-infarct patients (*n* = 17 before and 8 after).

Figure 4A,B present the level of IL-6 production in the three groups of patients at baseline and after 6 months of supplementation with EVOO. No significant differences or trend emerges between the three groups. Similarly, stimulation with LPS do not show significant differences. However there appears to be a higher potential for IL-6 secretion in dyslipidemic and post-infarct patients. The EVOO intervention did not significantly impact the LPS response of monocytes from different groups (Figure 4B). Similar results were found for IL-6 production by monocytes at baseline and after 6-month intervention with EVOO (result not shown). It is of note that, at the baseline (pre-intervention), even if LPS levels of IL-6 production do not differ significantly between groups, when accounting for the difference between steady state and stimulated state, a significant difference in IL-6 production is observed between healthy and dyslipidemic and post-infarct patients as well (Figure S3 Supplementary Data).

**Figure 5:**
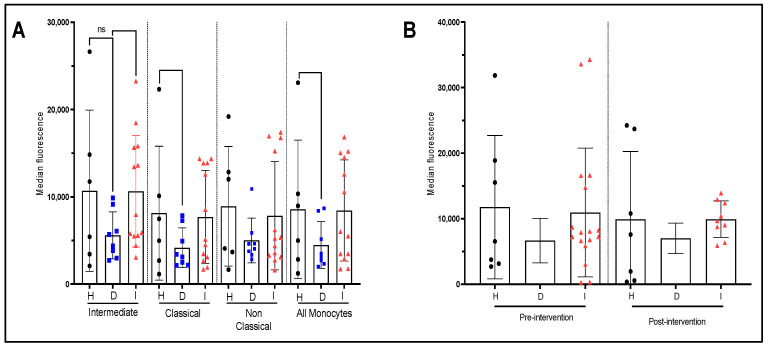
Production of IL-10 by monocyte sub-populations with and without LPS stimulation and effect of EVOO intervention. (**A**) Pre-intervention cytokine production by monocyte sub-population at quiescent state for each patient group. (**B**) Pre-and post-intervention cytokine production for the whole monocyte population after 4 h of LPS stimulation for each patient and group. The presented values represent measurement of the median fluorescence for IL-10. H: healthy participants (*n* = 7 before and 7 after). D: dyslipidemic patients (*n* = 12 before and 5 after) and I: post-infarct patients (*n* = 17 before and 8 after). ns = nonsignificant.

Conversely, monocytes at quiescence, either classical or intermediate, from dyslipidemic patients present a decreased production of IL-10 cytokine than healthy and post-infarct patients (Figure 5A). The trends persisted although the smaller sample made those differences not significant (*p* < 0.09) (Figure 5B). Interestingly, EVOO intervention did not significantly impact IL-10 production whether it be at quiescent state (results not shown) or stimulated (Figure 5B) and for both healthy, dyslipidemic, and post infarct patients.

**Figure 6:**
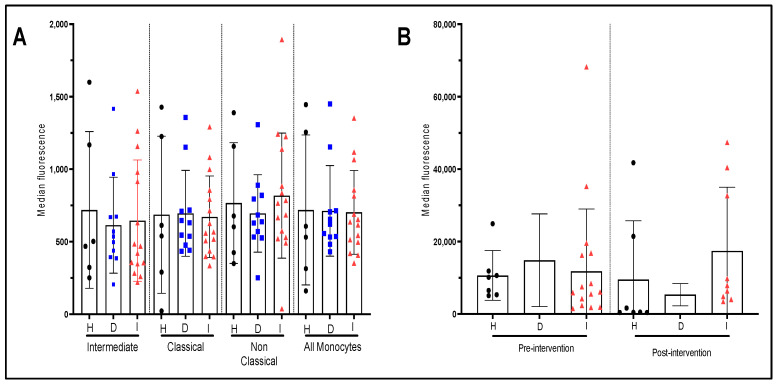
Production of TNF-α by monocyte sub-populations with and without LPS stimulation and effect of EVOO intervention. (**A**) Pre-intervention cytokine production by monocyte sub-population at quiescent state for each patient group. (**B**) Pre- and post-intervention cytokine production for the whole monocyte population after 4 h of LPS stimulation for each patient/group. The presented values correspond to the measurement of the median fluorescence for TNF-α. H: healthy participants (*n* = 7 before and 7 after). D: dyslipidemic patients (*n* = 12 before and 5 after) and I: post-infarct patients (*n* = 17 before and 8 after).

Figure 6A,B present the TNF-α production by monocytes of patients from the three groups of patients. As for IL-10, our results do show significant differences between groups in the capacity of their monocytes to secrete TNF-α at quiescence (Figure 6A) or after stimulation with LPS (Figure 6B). The EVOO intervention did not significantly modulate TNF-α production in monocytes whether stimulated by LPS or in non-stimulated state.

### 3.4. Plasma Cytokine Levels

**Figure 7:**
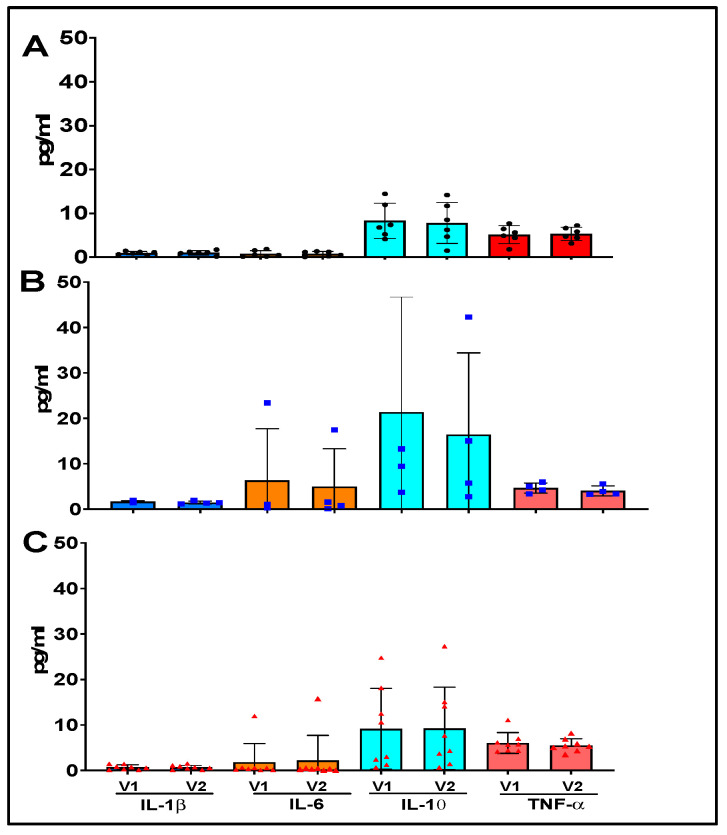
Cytokine concentration of IL-1β 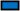, IL-6 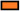, IL-10 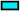 and TNF-α 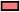 in the plasma. (**A**) Healthy control subjects, (**B**) dyslipidemic, and (**C**) post infarct patients at baseline and after 6-month EVOO intervention V1: Pre-EVOO intervention and V2: Post-EVOO intervention. H: Healthy (*n* = 7 at visit 1 (V1) and 6 at visit 2 (V2)). D: dyslipidemic patients (*n* = 12 at V1 and 6 at V2) and I: post-infarct patients (*n* = 17 at V1 and 8 at V2).

The plasma cytokine levels (IL-1, IL-6, IL-10, and TNF-alpha) were also analyzed. EVOO intervention did not significantly induce significant changes in these cytokines levels whether it be in healthy, dyslipidemic, or post infarct patients (Figure 7).

## 4. Discussion

Cardiovascular diseases, despite the numerous treatments, continue to generate a great morbidity and mortality. Inflammation, which plays a critical role in CVD, has been targeted by various interventions including the nutraceutical ones. In this paper we studied the effect of EVOO intake on monocyte phenotypes as well their cytokine production. Our results did not show any changes in either monocyte phenotypes or cytokine production by monocytes after 6-month of EVOO intake regardless of the cardiovascular risk level (healthy, dyslipidemic, or post-infarction).

Most studies in the literature on CVD point out that there could be differences in subpopulation distribution in infarcts and dyslipidemia, mainly that classical monocyte subsets would diminish whereas intermediate subsets increase [20]. In contrast, several other studies did not observe differences in monocytes subpopulation distribution whether it be in hypercholesterolemia [21], in CHD [22], or in CAD [23]. In the present study, our results did not show significant differences between monocyte subpopulation distribution among the three studied groups. However, we can notice that the tendencies are the same as the clinical studies showing significant phenotype differences—more intermediate and less classical monocyte in dyslipidemia and post-infarct patients compared to healthy control subjects. It is of note that all patients with dyslipidemia, in the present study, were free of any lipid-lowering treatment, which is quite different from other studies in which treated and non-treated patients as well as obese or diabetic patients are mixed [24,25]. Therefore, these results may have some clinical importance even if the statistical significance level was not reached.

Regarding cytokines production from dyslipidemic patients, our studied population presents a low and homogeneous levels of IL-10 compared to healthy control individuals, while there is a high diversity of pro-inflammatory signals due to numerous inflammatory cytokines and other pro-inflammatory mediators which are known to play a role in the development of atherosclerosis. In contrast, this cannot be said for anti-inflammatory signals where IL-10 and TGF-β are the main counterbalance for the local and systemic inflammation. The monocytes from the dyslipidemic group in our study could be in an inflammatory state, not because they have high levels of pro-inflammatory cytokines but because they present a homogeneous profile of low anti-inflammatory cytokines—IL-10. This finding correlates with the data from Collado et al. [26] where IL-10 levels in plasma and production by T-lymphocytes was significantly lower in primary hypercholesterolemic patients. This underlines the necessity not to study only the pro-inflammatory but also the anti-inflammatory mediators to establish a balance among them.

Surprisingly, the inflammatory status in dyslipidemic patients was more significant than that of post-infarction patients. However, it should be noted that our group of post-infarction patients are over-medicated in connection with their medical condition. On top of the statins, they have anti-inflammatories, antiplatelets, β-blockers, and many other medications. This set of drugs has a strong anti-inflammatory effect which could explain the fact that monocytes from post-infarct patients do not show an inflammatory activity. As a matter of fact, they show significantly less inflammation for the studied parameters than healthy controls with less IL-1β—a cytokine found to be impacted by statin treatment in most studies [27,28,29].

Stimulation of monocytes with LPS induced a comparable monocyte response regardless of patient group—healthy, dyslipidemic, or post-infarction. Thus, none of the patient groups showed an alteration in the response of monocytes to LPS stimulation (Figure S2). These results seem to be concordant that no changes in monocyte subpopulations were found in non-stimulated state. Even if we did not study specifically the trained immunity, our results seem to indicate that the monocyte of dyslipidemic and post-infarct patients from our cohorts are not in a hyper-activated state.

Surprisingly, EVOO intervention induced a significant increase in the IL-1β by monocytes of post-infarct patients when no other cytokines did. Since this increase in IL-1β did not happen concurrently with other inflammatory cytokines, this may suggest that the underlying transcription factor modulated by the intervention must not be the NF-κB pathway but rather via NLRP3 inducing the inflammasome pathway. It has been shown that an increase in IL-1β in the heart of post-infarct patients could have a positive effect by inhibiting ventricular remodeling/fibrosis [30,31]. Measurement of plasma IL-1β and other inflammatory cytokines (IL-6 and TNF-α) did not show significant change following EVOO intervention. Nevertheless, we should keep in mind that systemic inflammation is not always correlated to monocytic activity as it is only one of the numerous inflammation-modulating actors as cytokines producers also group lymphocytes, polymorphonuclear leukocytes (PMN), endothelial and epithelial cells, adipocytes, and connective tissue [32]. Moreover, when looking at the production of cytokines of all monocytes, it is hard to separate the effect of the distribution of the subpopulation into a more active phenotype in terms of cytokine secretion than the differences between monocyte subclasses cytokine secretion alteration.

We should note that clinical trials have not reached a consensus on the effect of EVOO on monocyte-inflammatory activity because the results obtained greatly vary between trials. The disparity of the results could be attributed to many factors like the quantity of EVOO used in the supplementation, as the absorption is dose-dependent [33], or the quality of the EVOO that includes the conservation conditions of the EVOO used (4 °C and protected from light). Moreover, although the cardioprotective effect of EVOO is well established, the results of a meta-analysis of prospective cohort studies showed that the beneficial effect of EVOO increases with the daily dose with no additional benefits beyond 20 g/day on CVD risk reduction and all-cause mortality [34].

In the circulation, phenolic compounds are only present in nanomolar quantities [35] and the increase in the antioxidant capacity-associated with phenolic compounds in the plasma stays noticeable only 1–2 h after phenolic compounds intake [36]. These polyphenols probably might affect individuals via cell signaling as it has been demonstrated in vivo [37] and not through systemic oxidative stress reduction. However, there is another parameter that needs to be considered for the postprandial effect, that is the digestion of the major components of EVOO and monounsaturated fats (MUFA). Some studies have shown that EVOO alone may affect the endothelium function during the postprandial phase [38,39]. Conversely, the beneficial effect of EVOO is significantly improved with an antioxidants-rich diet [40]. Olive oil might only be able to show its beneficial potential when taken concurrently with a Mediterranean diet, not a North American one [39,41]. All these considerations may explain why our EVOO treatment could not result into a concrete effect on monocyte subpopulations and cytokine production.

## 5. Conclusions

Our study demonstrates that there is no significant shift in monocytes subpopulation between the three patient groups studied as well as in the pro-inflammatory cytokine production. Small trends in dyslipidemic patients were found towards inflammatory monocytes subsets and in decreased anti-inflammatory cytokine production. The EVOO treatment alone could not be demonstrated beneficial for the monocyte homeostasis. Future studies are needed to evaluate how dyslipidemic patients could be targeted for more drastic anti-inflammatory treatment to decrease this risk factor to progress towards overt CVD. Moreover, elucidating the impact of EVOO on oxidative capacity of monocytes simultaneously with their inflammatory profile might be of interest.

Strengths of the study: The patient selection was very thorough with stringent criteria as our dyslipidemic patients are untreated and are not diabetic. Moreover, the cytokines observed encompass pro inflammatory as well as anti-inflammatory cytokines which allows a more complete assessment of inflammatory status of monocytes. The addition of plasma cytokines allows separating the monocyte inflammatory state from the systemic inflammatory condition. The patient compliance to the EVOO uptake was very closely monitored throughout the 6-month intervention.

Limitations of the study: We should note that our study has several limitations. The first limitation is the relatively small number of patients as the stringent exclusion criteria made recruitment challenging. Moreover, some participants dropped out of the intervention due to the follow-up requirements or for personal reasons. As a result, we had patients for whom we could not have data for the second visit (after 6 months of intervention with the EVOO). The second limitation is related to the absence of control over the concomitant diet of the enrolled subjects. The last limitation could be that these subjects were maybe more aware of their health by following a better lifestyle when deciding to take part in the study.

## Figures and Tables

**Table 1:** Anthropometric and clinical parameters of participants at different cardiovascular risk level (healthy, dyslipidemic, and post-infarct patients) at baseline and after 6 months of EVOO consumption.

	Pre-Intervention			Post-Intervention		
Groups	Healthy	Dyslipidemic	Post-Infarct	Healthy	Dyslipidemic	Post-Infarct
N (% m/w)	7 (67/33)	12 (16/84)	17 (69/31)	7	5	8
Age (years)	76.36 ± 5.21	73.40 ± 4.81	72.78 ± 5.70	79.4 ± 2.07	73.40 ± 4.81	72.78 ± 5.70
Height (cm)	166.70 ± 8.86	164.11 ± 6.22	165.30 ± 10.87	166.6 ± 7.23	164.32 ± 6.22	165.31 ± 10.87
Weight (kg)	81.46 ± 25.97	63.50 ± 12.63	75.92 ± 12.32	82.00 ± 11.91	64.50 ± 12.50	77.33 ± 12.19
BMI (kg/m^2^)	28.94 ± 7.47	23.48 ± 3.38	27.52 ± 1.82	29.58 ± 4.25	23.77 ± 3.23	28.07 ± 1.38
Waist (cm)	107.60 ± 27.37	86.75 ± 13.82	99.10 ± 5.29	102.81 ± 14.52	85.75 ± 12.95	99.89 ± 5.09
SAP (mmHg)	143.70 ± 19.31	133.33 ± 23.92	140.4 ± 20.03	140.22 ± 31.03	134.00 ± 6.83	129.40 ± 15.64
DAP (mmHg)	81.77 ± 15.14	78.51 ± 7.05	76.44 ± 4.04	80.61 ± 12.58	76.5 ± 2.38	78.67 ± 7.14
Lp(a) (nmol/L)	57.06 ± 71.59	87.55 ± 75.85	130.20 ± 85.34	80.81 ± 54.34	81.68 ± 74.18	125.7 ± 95.25
ALT (UI/L)	19.57 ± 6.10	17.25 ± 4.43	58.89 ± 70.42	15.63 ± 7.30	18.5 ± 4.2 *	36.33 ± 26.66
AST (UI/L)	20.29 ± 4.45	20.25 ± 1.89	43.11 ± 46.95	18.2 ± 5.07	22.67 ± 6.35	33.71 ± 23.64
CRP (mg/L)	1.76 ± 1.61	1.7 ± 0.89	2.26 ± 3.09	1.58 ± 1.24	1.78 ± 1.1	1.46 ± 1.08
TC (mmol/L)	4.88 ± 0.63 ^+-^	6.01 ± 0.42 *^+^	3.04 ± 0.52 *^-^	4.54 ± 1.21	6.39 ± 0.75	3.12 ± 0.49
TG (mmol/L)	1.21 ± 0.60	0.86 ± 0.14	1.17 ± 0.6	0.93 ± 0.20	0.83 ± 0.17	1.44 ± 0.98
C-HDL (mmol/L)	1.58 ± 0.49	2.06 ± 0.69 *	1.08 ± 0.23 *	1.49 ± 0.40	2.27 ± 0.72	1.08 ± 0.28
C-LDL (mmol/L)	2.75 ± 0.47 ^-^	3.56 ± 0.38 *	1.43 ± 0.54 *^-^	2.63 ± 0.96	3.74 ± 0.26	1.38 ± 0.45
TC/HDL	3.33 ± 0.90	3.23 ± 1.16	2.91 ± 0.75	3.13 ± 0.57	3.03 ± 0.94	2.98 ± 0.59
Non-HDL (mmol/L)	3.28 ± 0.54 ^+-^	4.27 ± 0.48 *^+^	1.96 ± 0.54 *^-^	3.05 ± 0.50	4.12 ± 0.31	2.04 ± 0.4
HbA1c (%)	5.58 ± 0.32	5.35 ± 0.45	5.74 ± 0.41	5.58 ± 0.37	5.35 ± 0.41	5.81 ± 0.62

SAP: Systolic arterial pressure. DAP: Diastolic arterial pressure, ALT: Alanine transaminase. AST: Aspartate transaminase. BMI: Body Mass Index. In Pre-intervention: ^+^ *p* < 0.05 between healthy and dyslipidemia, ^-^ *p* < 0.05 between healthy and post-infarct, * *p* < 0.05 between dyslipidemia and post-infarct. In Post-intervention: * *p* < 0.05 V1 & V2 of the same health status group.

## Data Availability

Not applicable.

## Supplementary Materials

The following supporting information can be downloaded at: https://www.mdpi.com/article/10.3390/nu15173819/s1, Figure S1: Gating strategy for identification of monocyte subsets.; Figure S2: Effect of EVOO on IL-1b production in dyslipidemic and post-infarct patients. IM: intermediate monocytes, CM: classical monocytes, NCM: non-classical monocytes, all monocytes. * *p* < 0.05.; Figure S3: Median fluorescence differential between LPS stimulated Classical monocyte and Classical monocytes at quiescence for each studied cytokine pre-intervention. (A) for IL-1β and IL-10. (B) for TNF-α and IL-6. Acronyms H represents Healthy. D represents Dyslipidemia and I represents the post-infarcts patient cohorts. * *p* < 0.05; Figure S4: Estimated Paired T-test for IL-1β. IL-10. TNF-α and IL-6. No significant differences where observed.
