# Supplementary material for: The Immunomodulatory Effects of a 6-Month Extra Virgin Olive Oil Intervention on Monocyte Cytokine Secretion and Plasma Cytokine Levels in Dyslipidemic and Post-Infarct Patients: A Clinical Pilot Study
Source: Nutrients. 2023 Aug 31;15(17):3819. doi: 10.3390/nu15173819 (PMC10489670; doi:10.3390/nu15173819)
Supplement: Supplementary file 1 [file nutrients-15-03819-s001.zip › nutrients-2569980-supplementary.pdf]

Article

The Immunomodulatory Effects of a 6-Month Extra Virgin Olive Oil Intervention on Monocyte Cytokine Secretion and Plasma Cytokine Levels in Dyslipidemic and Post-Infarct Patients: A Clinical Pilot Study

Adrien Zimmer [1], Alyanne Otrante [1], Nada Zoubdane[1], Michel Nguyen [2], Tamas Fülöp [1] and Abdelouahed Khalil [1,3,4,*]

Supplementary Materials:

Flow Cytometry analysis

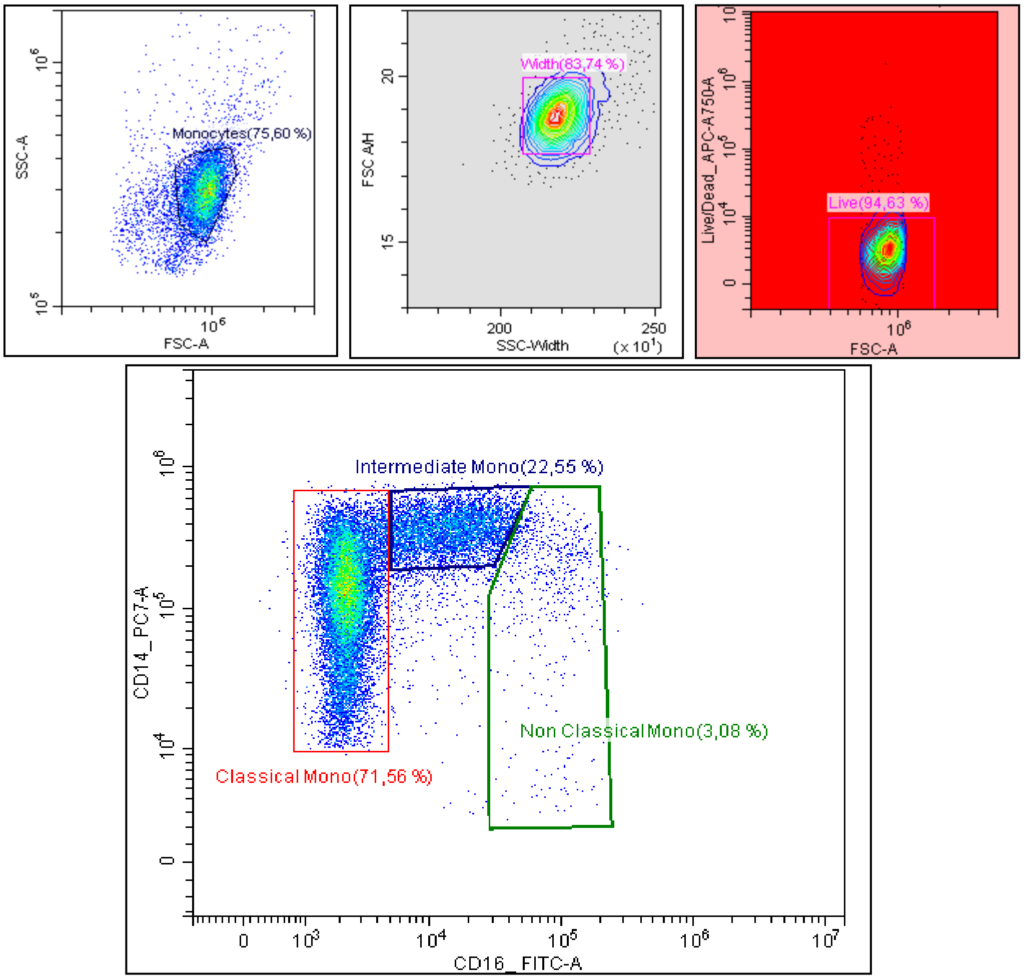

Figure S1: Gating strategy for identification of monocyte subsets.

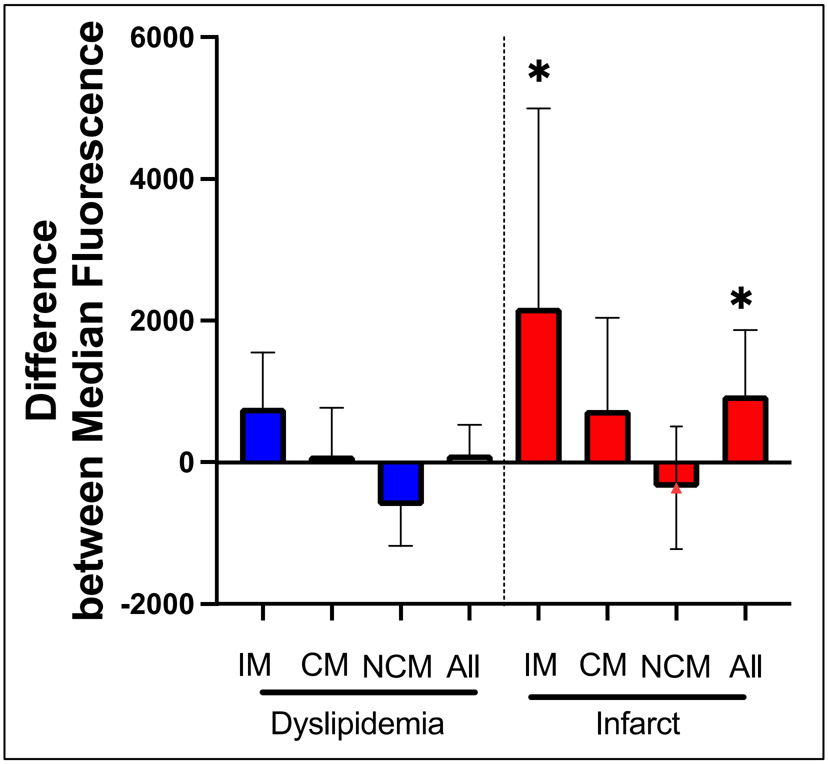

Figure S2: Effect of EVOO on IL-1b production in dyslipidemic and post-infarct patients. IM: intermediate monocytes, CM: classical monocytes, NCM: non-classical monocytes, all monocytes. * $p < 0.05$.

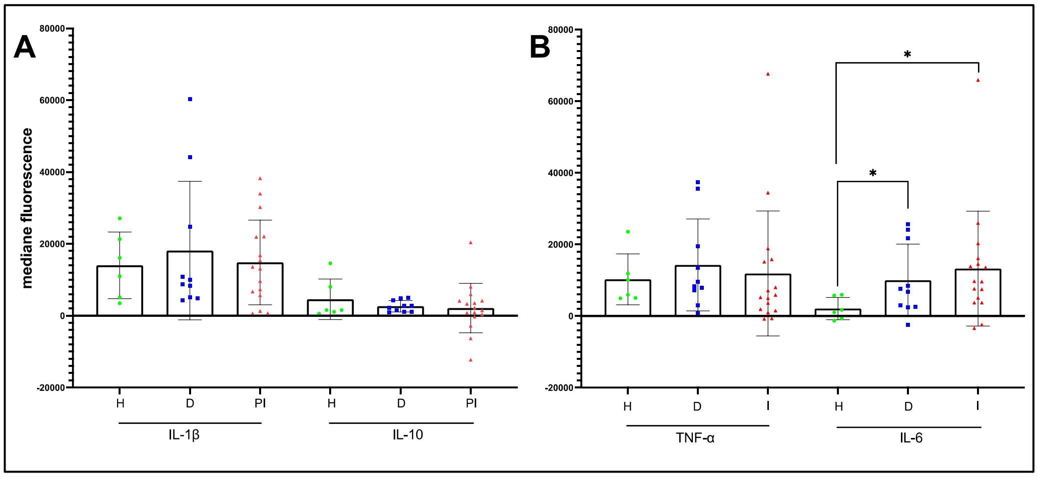

Figure S3: Median fluorescence differential between LPS stimulated Classical monocyte and Classical monocytes at quiescence for each studied cytokine pre-intervention. (A) for IL-1β and IL-10. (B) for TNF-α and IL-6. Acronyms H represents Healthy. D represents Dyslipidemia and I represents the post-infarcts patient cohorts. * $p < 0.05$

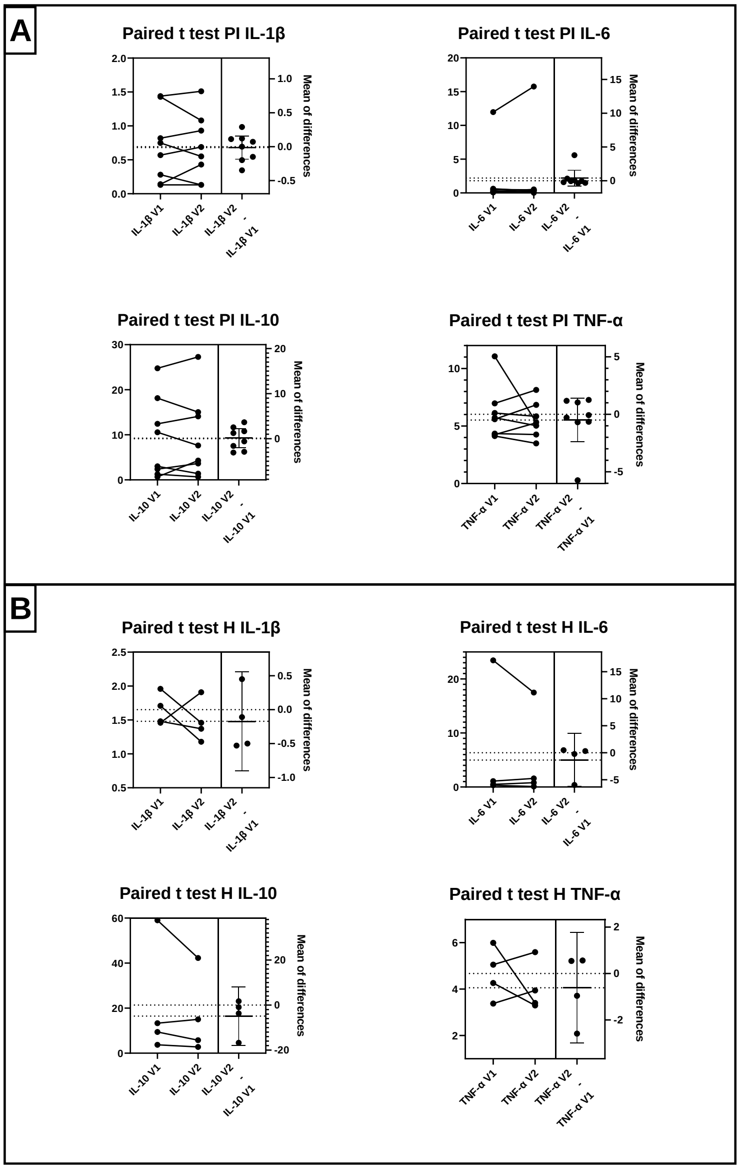

Figure S4: Estimated Paired T-test for IL-1β. IL-10. TNF-α and IL-6. No significant *differences where observed.*
